# South African Jewish Responses to COVID-19

**DOI:** 10.1007/s12397-021-09367-1

**Published:** 2021-04-13

**Authors:** Shirli Gilbert, Leah Gilbert

**Affiliations:** 1 Department of Hebrew and Jewish Studies, University College London, London, UK; 2 Department of Sociology, University of the Witwatersrand, Johannesburg, South Africa

**Keywords:** South Africa, Jews, COVID-19, Post-apartheid

## Abstract

Although the death rate caused by the COVID-19 pandemic in South Africa has thus far been much lower than initially feared, the economic and social impact has been severe. The country’s Jewish community, constituting 0.1% of the population with a median age of 45 years, has not escaped its effects. Organizations and individuals have nonetheless been able to mobilize a rapid and wide-reaching series of responses directed towards those most in need both inside and outside the community. The uniquely coordinated, energetic, and multipronged nature of these responses are attributed to robust communal infrastructure, strong community social capital, and the history of the Jewish community’s positioning in post-apartheid South Africa, alongside the perceived importance of health to collective well-being.

## Introduction

At the time of writing, South Africa was in 11th position on the Worldometer’s list of countries with the most COVID-19 cases in the world, with a cumulative total of 698,184 cases and 18,309 deaths (Worldometer 2020; Department of Health 15 October 2020).^1^ Although the death rate has thus far been much lower than initially feared, the economic and social impact of the pandemic has been severe. The country’s Jewish community has not escaped its effects, although the strength of communal infrastructure and resources (human, social, and financial) have facilitated a robust response directed towards those most in need both inside and outside the community.

President Cyril Ramaphosa was praised for his swift and decisive early action following the first reported case in South Africa on 5 March 2020 of a man who had recently returned from Italy (Department of Health 5 March 2020). On 15 March, a national state of disaster was declared in terms of the Disaster Management Act, with “urgent and drastic measures” taken including travel restrictions, the closure of some ports, limits on gatherings, and school closures (Presidency 15 March 2020). On 17 March, Ramaphosa convened the first meeting of the National Command Council on COVID-19 (Presidency 18 March 2020), and on 23 March, announced a lockdown with severe restrictions, with the South African Defence Force deployed to support the police in ensuring compliance. While restrictions were gradually eased from the beginning of May 2020, primarily for economic reasons, the major surge in infections came in July and August, leading to the imposition of new measures, including a reinstitution of the ban on alcohol sales and a curfew (Ramaphosa 2020). Restrictions were relaxed on Sunday, 20 September 2020.

Before COVID-19, South Africa was in its second recession in two years. The pandemic has impoverished the country further and deepened existing inequalities. Around one-third of those who had earned an income in February no longer did in April. The unemployment rate, already high, rose further as a result of the severe lockdown (Anon 18 July 2020). Reporting on 30 September on a nationally representative survey of the pandemic’s employment and welfare impacts, journalist Ferial Haffajee painted a bleak picture of South Africa’s post-COVID-19 economy, which she suggested resembled a “post-war landscape” with unprecedented levels of hunger and joblessness soaring over 50% (Haffajee 2020).

Numbering around 52,300 in 2019, the Jewish community in South Africa makes up less than 0.1% of the country’s population (Graham 2020).^2^ This size constitutes a significant decline from its peak, in the 1970s, of around 120,000. Emigration is primarily to blame, and the preponderance of younger people among emigrants accounts for the group’s median age of 45 years. The community is overwhelmingly urban, with the majority in Johannesburg (57%), and smaller centers in Cape Town (24%) and Durban (7%). South African Jews have long demonstrated very high levels of Jewish identification (Bruk 2005; DellaPergola and Tal 1978; Dubb 1977, 1994; Dubb and DellaPergola 1988; Kosmin et al. 1999), a point reinforced in a recent report by the Institute for Jewish Policy Research and the Kaplan Centre for Jewish Studies at the University of Cape Town, which surmised that “Jewish identity in South Africa may well be stronger, and more religious, than in either Australia or the UK” (Graham 2020: 35). At the same time, the majority of South African Jews also feel a strong sense of belonging to and identification with South Africa (Graham 2020: 71).

Because of the greater need among underprivileged communities in South Africa for services such as health, education, and welfare, more affluent communities—among them Jews—have historically relied more extensively on their own resources than might be the case in wealthier Western societies. A majority of South African Jews are well-off, or at least economically comfortable; however, over one-quarter (27%) of respondents to the 2019 survey said they were “just getting along,” and 3.2% described their situation as poor or very poor (Graham 2020: 86). Thus, despite an overall picture of relative wealth there is also a great deal of need, levels of which increased steeply during the pandemic. As a community with high levels of self-employment—a total of 26% of Jewish South Africans are self-employed, with the figure rising to as high as 39% in Durban (Graham 2020: 82)—the economic impact of COVID-19 has hit particularly hard. While a national financial relief scheme, the Solidarity Fund, was put in place to assist South Africans in need,^3^ evidence suggests that Jews have turned primarily to Jewish communal organizations, which have experienced a “drastic increase” in applications for assistance (Feinberg and Moshe 2020).

## Rapid Mobilization of Responses to COVID-19

The unique response of the South African Jewish community to COVID-19 must be understood firstly within the larger context of relationships between Jews and health. Scholarship suggests that Jews have a heightened concern for health relative to other groups (Shuval 1992; Dorff 2002). Jewish folklore is rich in sayings and proverbs pertaining to health and illness, and health is a frequent conversation topic, despite the fact that Jewish life expectancy is comparatively high (Staetsky 2020). Evidence indicates that Jews place a high value on health and are frequent users of medical services. Preservation of health may be viewed as an individually controlled survival mechanism in a community that has been continuously subjected to existential threats. Individual health and well-being is considered not solely a private affair but also the concern of the community as a whole (Gilbert 2009).

Jews in South Africa responded to the pandemic with a range of initiatives detailed in the pages that follow. The common theme among them is the speed and efficiency with which resources were mobilized. The community was able to do this in part because of its strong and highly centralized communal infrastructure. Unlike other Diaspora communities, in South Africa a great deal of emphasis has historically been placed on communal unity. A network of national institutions has long overseen the greater part of communal life, and most Jewish activities, whether religious or secular, take place within the framework of national coordinating bodies such as the South African Jewish Board of Deputies (SAJBD) and the South African Zionist Federation (SAZF). There is a well-developed network of Jewish schools, and synagogue affiliation is widespread (Dubb and DellaPergola 1988: 102; Graham 2020: 48). The Chief Rabbi is a central and largely unchallenged religious authority, despite the fact that many South African Jews practice a form of “non-observant Orthodoxy.” Such centralization has caused friction in other domains of Jewish life, and in recent years criticism has increasingly been voiced by Jews uncomfortable with what they perceive to be the mainstream community’s unwarranted control and its silencing of dissenting views (Gilbert and Posel 2021a, b). In the context of the pandemic, however, it allowed for an extremely well-organized and effective communal response. Responding early in the pandemic to the question of whether it was appropriate to criticize communal leadership or engage in “potentially fractious” debates, the former Cape South African Jewish Board of Deputies Executive Director, David Jacobson, responded that “there has been a silent agreement that communal issues that were once important have taken a back seat” (Brotman 2020).

In addition to its robust organization, South African Jewry’s response has also been facilitated by its ability to marshal community social capital, a particular form of social capital comprising institutions and associated informal networks that aim to contribute to the common good. Classic studies of social capital identify its three vital components as being social relations and networks, norms, and trust (Putman 1996). A recent study (Saukani and Ismail 2019) identifies spirituality and culture as an additional component. In many aspects of South African communal life, all of these components are clearly evident. Another recent study in the USA (Sadri et al 2018) demonstrates the positive role of social capital, such as strong personal and neighborhood networks, in aiding post-disaster recovery and community resilience.

The post-apartheid context is also significant. While Jews have historically been well represented in both antiracist political activism and nonpolitical welfare projects in South Africa, the mainstream community’s relationship to such work, particularly the former, before the 1990s was uneven and highly ambivalent (Shimoni 2003). Following the transition, communal investment in outreach has expanded significantly and is explicitly framed within a narrative highlighting the value and contribution of Jews to the new South Africa.

Taken together, the centrality of health, robust communal infrastructure, and strong community social capital against the background of the Jewish community’s particular positioning in post-apartheid South Africa helps to account for the uniquely coordinated, energetic, and multipronged nature of the community’s pandemic response.

Data for this article were collected by surveying a wide range of sources covering the period from March to October 2020, including all issues of the weekly *South African Jewish Report*, monthly *Cape Jewish Chronicle*, bi-monthly *Jewish Life* magazine, and quarterly *Jewish Affairs* journal; websites, emails, social media, and other public communications of major community institutions, the Office of the Chief Rabbi, and Jewish-led relief initiatives and organizations; and national and international press relating to South African Jewry, identified by keyword searches. The relevant content was analyzed using ATLAS.ti version 8.4.24.0. Activities were categorized by their initiators (community institutions, existing Jewish-led relief organizations, ad hoc Jewish groups and individuals), focus (health, finance, social welfare, religious life), and recipients. Cooperation between institutions and mobilization of existing infrastructure and resources was examined, as were framing narratives for the various types of activities.

## Inreach^4^

The SAJBD, the community’s umbrella representative spokesbody, was one of the first organizations in the country to provide medical information and advice on COVID-19. Emphasis was placed on scientific expertise, and the contribution of specialists within the Jewish community was exploited to its fullest potential. Professor Barry Schoub, an infectious diseases expert and founding Director of South Africa's National Institute for Communicable Diseases (NICD), served from the earliest weeks of the pandemic as an active medical advisor in a range of communal forums. [On 14 September 2020, Schoub was chosen to chair the government’s newly formed Ministerial Advisory Committee on vaccine development (Nordling 2020).] Additional experts in a range of medical fields, as well as experts in statistics, finance, behavioral economics, and other relevant subjects were identified and brought in as advisors to committees, to speak on webinars or on local community radio station ChaiFM, or to be interviewed for communal publications.

The communal response at the onset of the pandemic was swift and coordinated. Shortly after news of the first community case was received, the SAJBD convened an emergency meeting on 12 March including the leadership of major communal bodies from Johannesburg, Pretoria, Cape Town, and Durban, including the Chief Rabbi, representatives of the SAJBD (National, Gauteng, Cape, KwaZulu-Natal, and Pretoria), SAZF, the South African Jewish Board of Education, Union of Orthodox Synagogues, South African Union for Progressive Judaism, Community Security Organization, Hatzolah Medical Rescue, and South African Union of Jewish Students, among others (Hamodia 13 March 2020). Also attending the meeting were Schoub and Dr. Richard Friedland, chief executive of Netcare, one of South Africa’s leading private healthcare providers (Zagnoev 20 March 2020). Both men, joined later by additional medical experts from the Jewish community, were to play a significant ongoing role in shaping the Jewish leadership’s response.

Numerous observers remarked on the unprecedented nature of the cross-communal collaboration at this time. The Chief Rabbi wrote: “I have been overwhelmed at the coordinated and swift efforts of our leadership. It has been so affirming to be part of a dedicated team […] who are so dedicated to our community’s well-being” (Goldstein 20 March 2020, 20 March 2000). “We’ve never seen this level of unity in our community before,” said Zev Krengel of the SAJBD. “This could be our finest hour yet” (Miltz 20 March 2020).

At the meeting, subgroups were created to focus on specific issues, including welfare, education, and religion (Zagnoev 20 March 2020). Decisions were taken by the relevant organizations to preemptively close schools, synagogues, and old age homes, and to cancel all communal functions (Leibowitz 20 March 2020). There was widespread community support for this decision, despite a few synagogues insisting on remaining open until the official national lockdown began on 27 March 2020 (Feinberg 27 March 2020).

Following the meeting, a set of detailed communal guidelines, determined in consultation with experts, was made public, and an online Jewish community platform was launched. The platform, accessible on the SAJBD’s website and Facebook page, provided regular and up-to-date medical advice and communal guidelines, a regular podcast, and an interactive forum with Professor Schoub. Within a few weeks, the SAJBD reported that, in addition to proving a valuable resource for the community, the platform was also “increasingly being used by other communities as well” (Zagnoev 27 March 2020). Throughout the pandemic the SAJBD continued its work communicating with and educating the Jewish community, helping members to access psychological support, financial advice, and economic relief from government programs and banks, and repatriating South African Jews stranded abroad (SAJBD 19 March 2020, 28 April 2020, May 2020; Zagnoev 15 May 2020). As the pandemic progressed, streamlined cooperation among communal organizations continued to be evident, including regular meetings of communal leaders and experts, and a range of joined-up educational and relief initiatives.^5^

Communal organizations delivered an extensive range of support, spanning the gamut from physical and mental health to religious observance, home schooling, financial relief, food aid, and social welfare support. In part, as already stressed, this response relied on a well-established network of relief and fundraising organizations already in place before the pandemic struck. As journalist Tali Feinberg wrote following the disease’s peak in mid-August 2020, “We have done well because of our structures, and because of identifying deterioration quicker. There is no doubt that if our small community didn’t have these elements, the numbers would have been much higher” (Feinberg 14 August 2020).

The backbone of communal support is the Chevrah Kadisha, the oldest Jewish organization in Johannesburg and the largest Jewish welfare organization in Africa. The Chev, as it is popularly known, maintains a network of residential care facilities across the country, including orphanages, homes for the aged, and facilities for the intellectually disabled, and also offers financial assistance services. Before the pandemic, it was assisting around 3000 people each month with accommodation, healthcare, food, and education costs (Feinberg 10 July 2020). In May alone, it received more than double the usual number of applications (Feinberg 19 June 2020). The Chev’s work during the pandemic has focused primarily on the vulnerable residents in its care facilities, which were closed to the public as early as 13 March. COVID-19 protocols were rapidly implemented and a strict shielding policy instituted (Chevrah Kadisha 20 March 2020). In March, an online platform was launched providing up-to-date information for residents and their families (Chevrah Kadisha 25 March 2020), and regular communication was maintained in subsequent months. A range of ad hoc social services was introduced including a support service for over-70s requiring practical assistance or emotional support, and a WhatsApp group pairing volunteers with individuals in need (Chevrah Kadisha 30 June 2020, 23 July 2020). Chev social services also produced a series of COVID-19-related articles on topics such as building resilience during lockdown, staying mentally healthy, and the challenges of lockdown for single parents.

Another key organization was Hatzolah Medical Rescue (Hatzolah n.d.; SAJBD n.d.). Launched in Johannesburg in 1998, Hatzolah describes its main role as being “to care for people within the community who need our help, 24h, seven days a week.” Its volunteers are required to be “Torah-observant,” and the service is provided free to all Jewish people within the organization’s operating areas. In March 2020, the Hatzolah COVID-19 Wellness Programme was launched to monitor suspected or confirmed cases of the virus. With an emphasis on early intervention, the program provided daily tracking of temperature, heart rate, and oxygen saturation together with other health checks and check-ins. In addition to twelve full-time staff, the organization recruited dozens of volunteers, including counselors who offered psychological first aid (Miltz 10 July 2020). On 22 June, a parallel Wellness Monitoring Programme, developed alongside Hatzolah, was launched by the Community Security Organization in Cape Town (Myburgh 2020).

Several additional community organizations have adapted their work to address issues raised by the pandemic. Bikkur Cholim, a longstanding Jewish society for visiting the sick, was limited initially to sending care packages, but within a few weeks redirected its resources towards setting up a Home School Gemach, which offers free loans of computer supplies to help Jewish families with home schooling (Preskovsky 2020). Yad Aharon & Michael, which describes itself as “Johannesburg's leading, independent Jewish food fund,” makes weekly deliveries of “food parcels and dignity” to vulnerable members of the community (Friedman 4 September 2020). Koleinu SA, established in 2012 to confront domestic abuse and sexual assault in the Jewish community, increased its helpline hours and embarked on a social media campaign offering support to victims and helping parents put safeguarding measures in place for children (Koleinu SA 24 March 2020). The weekly *South African Jewish Report* hosted a series of webinars that attracted substantial audiences, showcasing Jewish expertise in a range of fields including medicine, business, and finance—some, but not all, pertaining specifically to the pandemic. The webinars also proved a useful platform to fundraise for communal organizations and initiatives (Maunder 15 May 2020).

Financial support was a key communal priority. The Rambam Charitable Trust, founded in 1995, provides interest-free loans to Jews across the community, assisting on average more than 150 people per year. When the pandemic began, the Trust created an emergency fund of R2.2m ($134,000), with an initial maximum loan amount of R10,000 ($605), later raised to R15,000 ($910). By the end of July, almost R3m ($182,000) of loans had been paid out to more than 275 families (Feinberg 24 July 2020; Feinberg and Moshe 1 May 2020; Rambam Charitable Trust n.d.). While the Rambam Trust focuses primarily on individuals, the Gesher Small Business Relief Fund, launched on 8 May 2020, is directed towards majority-Jewish-owned small- to medium-size businesses. Established by a group of Jewish philanthropists led by the Donald Gordon Foundation, Gesher is intended “to provide last resort, flexible term, interest free loans” in order to safeguard jobs and businesses and “enhanc[e] the stability of the Jewish community during this challenging period.” Gesher is run in conjunction with the Chevrah Kadisha and is led by over 50 volunteer professionals, representing the largest ever amount of capital—believed to be in the tens of millions of rands—made available to the South African Jewish community in a business-support loan scheme. By mid-July, the fund had received more than 400 expressions of interest and 140 applications, and granted 60 loans, benefiting 1000 employees and 10,000 direct dependents across the country (Feinberg 8 May 2020; Gesher n.d.; Anon 17 July 2020). Additional support was offered by the SAZF, whose iShuk initiative supports small Jewish businesses “by allowing community members to buy vouchers for their products and services and redeem them later” (iShak FAQs n.d.). Jews in South Africa were also able to apply to the Jewish Agency's COVID-19 Crisis Loan Fund, directed towards “already-struggling” communities in the diaspora (Jewish Agency for Israel n.d.).

With synagogues closed in mid-March 2020, religious life was severely curtailed. Some private *minyanim* (prayer quorums) continued to meet against the advice of communal leadership and rabbinate (Feinberg 12 June 2020), but for the most part religious activity was reconvened online, as was the case in religious communities across the globe. One notable exception was Sandringham Gardens, a home for the Jewish aged in Johannesburg which has its own synagogue. Thanks to the Chevrah Kadisha’s preemptive quarantining of the site, the synagogue became for a time the only space in the country where regular *minyanim* could still officially be held. The *minyan* soon began receiving requests to say *Kaddish* (a prayer for the dead that requires a *minyan* in order to be recited) on behalf of Jews in South Africa and around the world. They also performed baby-namings for Jewish families as far afield as Canada, the USA, Israel, Australia, the UK, and Uruguay (Moshe 27 March 2020; Shepherd 29 April 2020).

Rabbis across the country, and particularly the Chief Rabbi, worked actively to facilitate continued religious observance as well as provide spiritual guidance and inspiration for the community. Unsurprisingly, their messages to congregants and the wider community consistently emphasized the value of faith and prayer and encouraged Jews to observe the Sabbath and find new connections to their faith (Kievman 20 March 2020; Herrmann 27 March 2020; Widmonte 15 May 2020; Goldstein 13 September 2020). Grappling with the question of how to cope during the early weeks of the epidemic, Chief Rabbi Dr. Warren Goldstein wrote: “At a time like this, we need to deepen our faith and connection to our creator, turning to Him with our heartfelt prayers, knowing that He cares and He listens, and that the world, and our lives, are in His loving hands” (Goldstein 20 March 2020, 20 March 2000). Goldstein also announced the launch of a community WhatsApp group “to connect in real-time and offer timeless guidance, inspiration and support” (Goldstein 21 July 2020). Just before Passover, in early April, the Chief Rabbi presented *All Together Now: A Unity Haggadah Companion*, a “compendium of insights and inspirational ideas” by rabbis and rebbetzins [rabbis’ wives] across the country (Goldstein 7 April 2020). A counterpart was produced for Shavuot, the second festival celebrated in lockdown, in late May (Goldstein May 2020). The South African arm of worldwide Hasidic movement Chabad provided online resources on a range of topics, including observance of Shabbat and festivals during the pandemic, spiritual guidance and upliftment, and practical guidance for home schooling (Chabad House Johannesburg n.d.).

## Outreach

In addition to extensive inreach work, an extraordinary array of initiatives for food aid and humanitarian relief directed towards broader South African society was launched by Jewish institutions, individuals, and informal groups during the first seven months of the pandemic. Before COVID-19 there was already a well-established network of organizations focused on underprivileged communities. Such organizations were able to adapt quickly and direct their activities where they were needed most. Afrika Tikkun, a longstanding Jewish-led humanitarian organization, was involved in widespread initiatives, working in partnership with the South African supermarket chain Pick n Pay, Krost Steel, and Stallion Security. It was also selected as one of the distributing agencies for the government’s Solidarity Fund, providing tens of thousands of food packs (Lubner 24 April 2020). SA Harvest, also working in food aid, scaled up its operation in response to the mass unemployment and resulting food insecurity caused by the pandemic (SA Harvest n.d.). Boikanyo: The Dion Herson Foundation, active in poverty alleviation work in Johannesburg since 2011, expanded its work during lockdown to distribute a highly fortified cereal called ePap to impoverished communities across the country (Feinberg 12 June 2020b). The Nashua Children’s Charity Foundation, which was assisting dozens of orphanages, early childhood development centers, special-needs and outreach centers pre-pandemic, stepped up its work significantly and teamed up with members of the Jewish community to organize major food drives. The Foundation's founder, Helen Fraser, explained that her Jewish identity was a “driving factor” in her work, and noted that she “just wanted to do *tzedakah* [charity] and *chesed* [kindness]” (Feinberg 12 June 2020c). Afrika Awake, a non-profit organization formed in 2013 to address growing xenophobic violence against non-nationals in South Africa, expanded its work to provide food aid in the Tembisa and Alexandra townships near Johannesburg (Moshe 8 May 2020). The Angel Network, started by Glynne Wolman in 2015, grew its scope dramatically, moving to spending 90% of its funds on food parcels and ePap where they had not previously dealt with food at all. “Before COVID-19, we were assisting 50 non-governmental organizations, outreach centers, safe havens, and orphanages, who together numbered about 40,000 people,” explained Wolman. “Currently, we are trying to help more than 90 organizations which equates to helping well more than 100,000 people” (Feinberg 12 June 2020d).

The support of the SAJBD has been an important enabling factor for the expansion of Jewish humanitarian work. From the first weeks of lockdown, the Board was working to facilitate direct links between donors and communities, mobilizing existing networks to allow streamlined relief to be delivered to those in need. Central to this was the Collective Action Network (CAN) initiative, “in which ad-hoc volunteer associations focusing on emergency poverty relief are put together within existing community structures or networks” (Zagnoev 8 May 2020). The SAJBD partnered with the Angel Network to expand the CAN initiative from the Western Cape into Gauteng Province (where Johannesburg is located), and during lockdown many small Jewish-led groups were formed across the country under the CAN umbrella (Kahn July 2020). By early May, an estimated 40 of 55 CANs operating in Johannesburg were being run by members of the Jewish community (Miltz 1 May 2020). CAN is lauded as being “a genuine ground-up initiative, operating through community partnerships between suburb, informal settlement, and township” (Zagnoev 1 May 2020). Small groups of friends or neighbors organize local collections of donations, including food, toiletries, and non-perishable goods, and are then assisted with distributing the goods to appropriate recipients. On 14 May, the SAJBD announced that it had created a R9 million fund to help feed communities most affected by the pandemic, supported by funds from Jewish organizations and individuals (SAJBD 14 May 2020). Money from the fund was allocated and disbursed, in partnership with the Angel Network, to CANs as well as a range of other relief initiatives, Jewish and non-Jewish, including the Save a Soul project and Ripples for Change (Zagnoev 15 May 2020, 12 June 2020; Moshe 10 July 2020; SAJBD 13 July 2020; Kahn July 2020).

Also within existing communal structures, the Union of Jewish Women, which has since its founding in 1931 been committed to welfare work in the Jewish as well as broader South African communities, has been inundated with calls for support nationwide. Building on decades of experience and robust networks, it has been able to deliver food and food vouchers, clothing, blankets, educational materials, and nappies (diapers) to thousands of people in urban and rural areas across the country, and forged partnerships with local schools and organizations (Anon 29 May 2020).

Numerous ad hoc, local projects were also initiated in response to the pandemic. In early May, for example, twenty-something Capetonian photographer and filmmaker Chad Nathan launched his “Raising Hope” campaign, which sought to raise funds for organizations involved in emergency relief work. The campaign attracted widespread support and raised almost six times its initial target: at the time of writing, over R560,000 ($34,000) (Nathan 2020; Nathan n.d.). Fingertips of Africa, founded by businessman Yehuda Lazarus, supports vulnerable individuals and families in the Johannesburg area (Zagnoev 12 June 2020). A group of friends in Cape Town formed a group called One Bag Full, which collects nonperishable items and raises money for food for distribution in the Langa township. The group raised in excess of R120,000 ($7000) and delivered more than 30,000 meals. Echoing the remarks of many other Jews involved in relief efforts, the founders emphasize the extent to which their Jewish identity has driven their work: “Our Jewish and [local Jewish school] Herzlia upbringing encouraged awareness of *tzedakah* as well as giving back to those less fortunate. This was further engrained in us by our parents, and has made us conscious of what we have and many don’t have. From what we have seen during this bizarre time, there is a real Jewish spirit of caring and providing for others” (Feinberg 15 May 2020). Some inventive fundraising drives—including online boxing and art classes—were launched by teenagers and younger children, with funds donated to local charities (Leibowitz 8 May 2020; Leibowitz 15 May 2020; Staff Reporter 29 May 2020).^6^

## Discussion

Throughout the pandemic, politicians and leaders around the globe have offered narratives of unity to raise morale and assure their populations that “we will get through this together.” The leadership of South Africa has done similarly. “We are responding as a united nation to a common threat,” declared President Cyril Ramaphosa on 15 March 2020. “This national emergency demands cooperation, collaboration and common action. […] All the institutions of the state will be mobilized to lead this effort, but, if we are to succeed, every company, trade union, NGO, university, college, school, religious group and taxi association will need to play its part” (Presidency 15 March 2020). Ramaphosa’s rhetoric has emphasized not only solidarity, but also the importance of mobilizing all sectors of society to fight the pandemic.

Narratives of unity have also been espoused by the South African Jewish community, although in subtly differing ways. The messages emanating from the Office of the Chief Rabbi, which have met with widespread support, have been directed toward the Jewish community and its connections with other Jews in the diaspora, rather than the larger South African social context. “What has been so heartwarming throughout this challenging period is our communal unity,” said Chief Rabbi Dr. Warren Goldstein following the second meeting of the cross-communal leadership in late June. “All the different parts of our community—welfare and security organizations, schools, and shuls—have worked together for the common good. It’s this spirit of partnership, this unity of purpose, that gives us the strength and motivation to confront any challenge. Our unity is the heart of who we are as South African Jewry” (Miltz 26 June 2020: 3). Describing the motivations for setting up the Gesher Fund, Goldstein explained that it was “founded on a core Torah value: that we are here on this earth to help each other,” and stressed the importance of “com[ing] together as a community” to address urgent Jewish needs (Feinberg and Moshe 1 May 2020; Goldstein 8 May 2020; Kievman 22 May 2020). In “An open letter from the Chief Rabbis of the world,” published before Passover in early April, Goldstein joined the Chief Rabbis of Israel, France, Moscow, Argentina, the UK, Russia, Brussels, and Rome in calling for Jews around the world to join together in comfort and strength: “This is a time for us to rally together in unity. This is a time for us to be together—to welcome Shabbat in together—as one people with one heart” (Anon 3 April 2020). Ahead of the festival of Shavuot, Goldstein initiated a second call to action. In a letter penned together with the Chief Rabbis of Israel, Russia, France and Argentina, Goldstein recalled the “powerful moment of Jewish unity” at the foot of Mount Sinai, when Jews received the Torah. “These are challenges we're all facing, no matter who we are or where we come from, and we saw it as a crucial time for Jews around the world to set aside our differences and overcome conflicts, both communal and personal” (Judah 27 May 2020). There have been other similar transnational expressions of Jewish unity from both religious and lay leadership, offering affirmative messages of hope, solidarity, and optimism.

The approach of the SAJBD has been more multifarious in its focus. The Board was central to the internal Jewish communal response, as discussed above. At the same time, in its rhetoric it has repeatedly emphasized its commitment to broader South African society, aligning itself with Ramaphosa’s narratives of national unity. Referring to the R9m SAJBD relief fund announced in May, for example, National Chairman Shaun Zagnoev wrote:

The core mandate of the South African Jewish Board of Deputies (SAJBD) is to safeguard the rights and promote the welfare of South African Jewry, but there are times where, as the community’s representative body, we involve ourselves in matters of wider national concern. […] Today, the overriding challenge facing the country is to provide sufficient food to those unable to generate any kind of income due to lockdown conditions to sustain themselves and their dependents until the crisis has passed. The Board has been devoting much of its time and resources to making a difference in this area either through its own projects or by working with and supporting other Jewish-led upliftment initiatives. As our National Director Wendy Kahn stresses, in addition to seeing to the welfare of our own community, we need to be concerned about the real crisis of hunger in the greater society (Zagnoev 22 May 2020).

Zagnoev reiterates here the SAJBD’s “core mandate” of safeguarding and supporting Jews, a principle that dates back to the organization’s establishment in the early twentieth century, and that also—not without controversy—explained its prioritization of Jewish communal welfare during the apartheid period. In addition, however, Zagnoev stresses the equal importance, at this critical moment, of the community looking beyond its boundaries. This emphasis clearly provoked some disquiet, evident in the lively debate that greeted the fund’s announcement on the SAJBD’s Facebook page. “It's wonderful to assist,” commented one Jewish observer,

But how about charity begins at home. So many of our own community are in dire straits and the chev [Chevrah Kadisha] can only do so much. That 9 million could support Jewish schools, families, medical needs etc etc. No one in this country looks out for us and the Jewish owned businesses don't qualify for government assistance due to BBEEE [Broad-Based Black Economic Empowerment] requirements. So why are we so fast to use our resources before our own are safe!!! (SAJBD 14 May 2020).

Numerous similar comments were posted, and some Jews working in outreach initiatives have likewise reported being asked why they don’t support the impoverished within the Jewish community first (Moshe 8 May 2020).

Such views have also been robustly challenged, however, and evidence suggests that community support for both inreach and outreach initiatives has been generous and widespread. The rabbinate, too, has been mindful of having “a positive impact on wider South African society” in its relief work (Feinberg 8 May 2020). The day after the announcement of the R9m fund, the SAJBD issued a clarification, explaining that the donation had been earmarked expressly for the objective of “provid[ing] food for impoverished South Africans with no food as a result of the recent lockdown,” and that they “could not be utilised for any other purpose.” The Board also reminded readers that “Throughout the COVID crisis we have worked with our fellow communal organizations to ensure that the Jewish community is also supported” (SAJBD 15 May 2020).

The explicit rhetoric of outreach can be understood, in part, against the background of apartheid history and the thorny question of Jewish communal responses. Although there has not yet been a full reckoning with the apartheid past either among Jews or in broader society, the Jewish communal leadership is sensitive to accusations—heretofore leveled more by fellow South African Jews than anyone else—that they did not do enough to resist the racist regime. In what might be seen as a nod to this history, Zagnoev wrote in mid-June, a few weeks after the fund’s launch: “We applaud the life-saving work that is being done by Jewish organizations and individuals in all parts of South Africa, and are proud to be able to participate in it. Much remains to be done, but when looking back on this difficult and traumatic period, we know at least that at the moment of great crisis in our country’s history, South African Jewry hasn’t been found wanting” (Zagnoev 12 June 2020).

Perhaps more significantly, the enormous challenges posed by the pandemic have also heightened existing feelings of precariousness and vulnerability. For despite the robustness of the community’s infrastructure and its still considerable resources, there are concerns about its long-term health and prospects. On 19 June, the Chev was forced for the first time in its 132-year history to call for emergency financial support. Its work in both residential care and financial assistance—sectors especially impacted by the pandemic—left it severely exposed, and with almost no state support and overwhelming reliance on private donor funds, it was placed under unprecedented strain (Feinberg 19 June 2020). Following the launch of the Chev’s Emergency Relief Fund, the United Jewish Campaign and Durban Jewish Social Services, Jewish fundraising bodies in Cape Town and Durban, respectively, followed suit (Klawansky 29 June 2020). Jewish emigration from South Africa is ongoing, and indications are that it will increase in the coming months (Feinberg 21 August 2020), shrinking a community already in demographic decline. High rates of self-employment, as noted earlier, have meant that Jewish individuals have been hard-hit. Adam Mendelsohn, Director of the Kaplan Centre for Jewish Studies at the University of Cape Town, postulates that the economic impact on largely self-funded Jewish institutions may also significantly shift the communal landscape (Feinberg 11 September 2020). Although COVID-19-related antisemitism remains very limited in South Africa, there has been considerable vigilance about its potential to surface, particularly given its appearance elsewhere. All this is against the backdrop of internal rifts in the community that have long been evident and that have begun to surface more insistently in recent years, particularly around attitudes toward Israel (Gilbert and Posel 2021b). Given the complex challenges that need to be juggled, it is thus perhaps unsurprising that efforts are being made to mitigate communal disagreements, at least in the short term, in the interests of streamlined relief efforts and collective unity.

## Conclusion

Several interrelated factors help to explain the robustness of the South African Jewish community’s response to the COVID-19 pandemic. That the crisis pertains to health is key, tapping into deep-seated anxieties about individual and collective well-being. Alongside fears about the strength and future of communal life is consciousness of the plight of the mass of underprivileged South Africans, particularly against the background of the apartheid past and Jews’ historical positioning in the South African racial order. That consciousness is combined with a genuine and longstanding impulse to charitable work both inside and outside the community, as well as a deep sense of belonging and commitment to larger South African society. The strength of these combined motivations has animated the mobilization of an impressive range of initiatives across a wide scope—health, welfare, finance, and spiritual life—addressing the needs of both Jewish and broader South Africa, and facilitated by an efficient centralized infrastructure and strong community social capital.

At the time of writing, the pandemic is far from over. The community remains highly vigilant, and coordinated leadership continues to be delivered by the SAJBD, the Office of the Chief Rabbi, and the Chevrah Kadisha, together with other organizations and in partnership with Jewish experts. Some cracks, however, are already beginning to show. The extent to which it will be possible to retain the strength and coordination of these responses as the pandemic’s severe effects persist remains to be seen.

## Footnotes

1 Comparisons of estimated weekly excess deaths in South Africa with the number of official COVID-19 deaths suggest that the latter are significantly under-reported (South African Medical Research Council n.d.). Detailed data are only made available for provinces and local districts, and no public data are available for specific social groups. Some data relating to Jews in South Africa have been compiled by Hatzolah in Johannesburg and the Community Security Organization in Cape Town, but these are not comprehensive data on the Jewish community.

2 The Department of Statistics estimated South Africa’s population on 9 July 2020 at 59.62m. http://www.statssa.gov.za/?p=13453 (accessed 8 October 2020).

3 The Solidarity Fund, established on 23 March 2020, is a public benefit organization with a mandate to support the national health response, contribute to humanitarian relief efforts, and mobilize South Africans in the fight against COVID-19. Data are not available to determine the extent to which assistance has been requested from, or allocated to, Jews. https://solidarityfund.co.za/about/ (accessed 13 October 2020).

4 The terms “inreach” and “outreach” are used by the South African Jewish community to distinguish between initiatives directed towards members of the Jewish community and those intended for non-Jews, respectively.

5 A second, virtual meeting of cross-communal leadership was held in the last week of June 2020, attended by Chief Rabbi Dr. Warren Goldstein and representatives of communal structures and community organizations, as well as Jewish experts in the field of infectious diseases (Miltz 26 June 2020).

6 An overview video of Jewish-led organizations involved in relief during the pandemic is available at https://www.excelacademyinc.com/amazing-sa (accessed 5 October 2020).
